# Isolation, characterization, and application of lytic bacteriophages for controlling *Enterobacter cloacae* complex (ECC) in pasteurized milk and yogurt

**DOI:** 10.1007/s12223-023-01059-7

**Published:** 2023-05-15

**Authors:** Mohamed A. Nasr-Eldin, Eman Gamal, Mahmoud Hazza, Sabah A. Abo-Elmaaty

**Affiliations:** Department of Botany and Microbiology, Faculty of Science, Benha University, Benha, 13511 Egypt

**Keywords:** *Enterobacter cloacae* complex, Bacteriophage, Biocontrol, Milk, Yogurt

## Abstract

Reducing bacterial pathogen contamination not only improves overall global public health but also diminishes food waste and loss. The use of lytic bacteriophages (phages) that infect and kill bacteria could be a beneficial tool for suppressing bacterial growth during dairy products storage time. Four *Enterobacter cloacae* (*E. cloacae*) complex isolates which were previously isolated from contaminated dairy products were used to identify lytic phages in wastewater. Phages specific to multi-drug resistant (MDR) *E. cloacae* complex 6AS1 were isolated from local sewage. Two novel phages vB_EclM-EP1 and vB_EclM-EP2 were identified as myoviral particles and have double-stranded DNA genome. Their host range and lytic capabilities were detected using spot test and efficiency of plating (EOP) against several bacterial isolates. The phages had a latent period of 30 min, and a large burst size of about 100 and 142 PFU/cell for vB_EclM-EP1 and vB_EclM-EP2, respectively. Both phages were viable at pH ranging 5–9 and stable at 70 °C for 60 min. The individual phages and their cocktail preparations (vB_EclM-EP1 and vB_EclM-EP2) reduced and inhibited the growth of *E. cloacae* complex 6AS1 during challenge test in milk and yogurt samples. These results indicate that the *E. cloacae* complex-specific phages (vB_EclM-EP1 and vB_EclM-EP2) have a potential application as microbicidal agents in packaged milk and milk derivatives during storage time. In addition, our environment is a rich sources of lytic phages which have potential use in eliminating multidrug-resistant isolates in food industry as well as in biocontrol.

**Supplementary Information:**

The online version contains supplementary material available at 10.1007/s12223-023-01059-7.

## Introduction

*Enterobacter* is a facultative anaerobic Gram-negative bacillus and has become one of the major genera in *Enterobacteriaceae* since its first description in 1960. It usually lives in almost any climate, including natural intestinal flora, stools, plants, water, insects, and food (Liu et al. 2016). *Enterobacter cloacae* (*E. cloacae*) are considered one of the most important genus species frequently isolated from clinical samples and food products (Farmer et al. 1980; Mezzatesta et al. 2012; Morozova, et al. 2021). Extended-spectrum β-lactamase (ESBL) genes of *E. cloacae* confer resistance to most β-lactam antibiotics, including extended spectrum (i.e., second and third generation) cephalosporins (ESCs) and monobactams which make their treatment difficult (Annavajhala et al. 2019; Davin-Regli et al. 2016).

Milk and milk derivatives in many parts of the world form a crucial part of human diet and are suitable environment for numerous microorganisms due to its high nutrient content, near-neutral pH, and high water activity (Frank 1997; Nascimento et al. 2022). Microbial spoilage occurs attributed to the chemical composition of dairy products such as pasteurized milk, butter, ice cream, and yogurt. Yogurt comes after processing and fermenting milk, since it provides microbes with rich nutrients. Exposure to microbial contamination may occur during manufacturing, storage, and transport without appropriate sanitary procedures and temperature control can quickly deteriorate it and therefore become rejected for human consumption (Mohammed and Abdullahi 2015).

Contamination of packaged milk after heat treatment may happen due to a failure of the cleaning and sanitation program (Boor and Murphy 2005). Typical spoilage sources are damaged packaging, insufficient packaging sterilization, or improperly cleaned processing equipment (Fernandes 2009).

*E. cloacae* isolates have been previously isolated from contaminated dairy products in Egypt (Sobeih et al. 2020). Several approaches are used to help improve the safety of our foods. A significant number of different antimicrobial treatments of food products include potential changes in the properties of food and the risk for contamination in food (physical and chemical treatment). These treatments must be limited in order to minimize both risks, which limits their efficacy.

In order to overcome this problem, many therapies can be used in a series, called the “multiple hurdles” approach to decontamination (Sofos and Smith 1998; Bacon et al. 2000; Moye et al. 2018). Moreover, some therapies are focused on “normal” antimicrobials, such as plant extracts or microbial products (for example, bacteriocins), which allow food microbial ecology to be manipulated. In addition, with the current rise in antimicrobial resistance to many antimicrobial agents, the utilization of bacteria-eating viruses termed as bacteriophages (phages) have been recommended. Since the phages are self-replicating in the host bacteria then cause lysis, they aid in elimination of bacterial pathogens that cause food spoilage (García-Anaya et al. 2020; Nascimento et al. 2022). The use of phages has recently sparked an increased interest in this regard both from investigators and from manufacturers (Hudson et al. 2005; Cristobal-Cueto et al. 2021). Phages are bacterial viruses which are wide spread in the world including in food since they are part of the natural food microbiota (Brüssow 2005). There is at least one phage for virtually every bacterial species, which can directly invade this specific bacterial population and eventually kill it (Egido et al. 2022).

According to the nucleic acid type, the phages are divided into group I double-stranded DNA viruses, group II single-stranded DNA viruses, group III double-stranded RNA viruses, and group IV single-stranded positive-stranded RNA viruses. Currently, the majority of phages found belong to double-stranded DNA viruses, most of which are tailed phages (Wang and Zhao 2022). The two main characteristics of tailed phages include capsids that wrap genetic materials in the form of DNA or RNA and tails of different sizes in different phages.

Phages can be divided into two types, lytic and lysogenic phages. After the lytic phage infects the host, it begins to enter the lytic cycle; the phage hijacks the cellular machinery of the bacteria, shutting off the expression of host genes and achieving the replication of its genome and the expression of its own genes (De Smet et al. 2017).

Phages are capable of killing multidrug-resistant (MDR) bacteria and reducing the non-desirable effects produced by chemicals on food (Cristobal-Cueto et al. 2021). The use of phages to control foodborne pathogens has been explored in dairy products (García-Anaya et al. 2020; Esmael et al. 2021; Nascimento et al. 2022).

There are seventeen Enterobacter phages which possess the myovirus morphotype (Morozova et al. 2021). Here, we isolated two phages belonging to myoviruses morphology that infect dairy *Enterobacter* isolates. Phages are extremely promising to be substitutes to conventional antimicrobials in foodborne pathogens. There is a constant need to characterize new phages in order to assess their efficacy and safety, as biocontrol agents both in vitro and in vivo (Nale and Clokie 2021). In order to extend the shelf life of milk and milk products with the use of advanced storage technologies, there is need to consider an effective method to reduce food waste and losses. Manufacturing cocktails composed of several different phages with the broadest possible spectrum of antibacterial activity seems to be a relevant approach for success of using phages in food (Wójcicki et al. 2019; Tan et al. 2021; Rogovski et al. 2021). However, cases of utilizing virulent phages to combat *E. cloacae* complex that are usually associated with dairy products are rarely seen.

Here, we partially characterized *E. cloacae* complex infecting phages based on their morphology, growth kinetics, host range, and stability at different pH and temperature and bacterial reduction assay. These are crucial criteria to identify potential phages for biocontrol application (El-Dougdoug et al. 2019). In addition, we evaluated their effectiveness against *E. cloacae* complex in milk and yogurt. In this paper, we also have provided evidence that these phages were able to lyse and kill the MDR *E. cloacae* complex bacterial isolate in the milk and yogurt, allowing us to conclude that these newly isolated phages would have the potential to control the growth of these bacteria in milk and yogurt under different temperatures to improve food safety.

## Material and methods

### Bacterial isolates

Four *Enterobacter* spp. isolates used for phage screening were isolated from contaminated packaged milk and yogurt samples at different markets in Benha City, Qalyubia Governorate, Egypt. They were tentatively identified using biochemical tests (catalase test, methyl red test, oxidase test, Vogues-Proskauer test, indole test, urease test, and sugar fermentation tests (Stephan et al. 2008) (Table S1). *Enterobacter* spp. isolates were confirmed by VITEK® 2 COMPACT automated instrument for ID/AST testing (BioMérieux, Marcy-L’Etoile, France) at Reference Microbiology Lab., Water and Drainage Station, Shubra Al Khaimah, Qalyubia Governorate, Egypt, and were identified as *Enterobacter cloacae* Complex. The susceptibility testing for *E. cloacae* complex isolates to twelve different antibiotics was performed by disk diffusion method (CLSI 2018). *E. cloacae* complex isolates were resistant to at least 50% of the tested antibiotics and were consequently recognized as multi-drug resistant (MDR) (Table S2, Fig. S1).

### Isolation and propagation of phages

Phages were isolated from sewage, milk, and whey samples that were collected from Benha City, Qalyubia Governorate, Egypt. Phages were detected in sewage by spot test and plaque assay according to Adams (1959) and Jurczak-Kurek et al. (2016) for *Enterobacter* spp. The obtained phage plaques were purified through picking the phage plaques 2–3 times, followed by growing through the double-layer agar technique. The phage titer in each lysate was expressed as plaque-forming units per milliliter (PFU/mL). Phage lysates were propagated on the corresponding bacterial host strain (*E. cloacae* complex 6AS1) to obtain high titer stocks. Crude phage lysates were centrifuged at 4000 × *g* in EBA 12 R centrifuge (Hettich lab technology, Germany) for 20 min. Supernatants were filtered through 0.22-µm filter or 0.45-µm pore-size syringe filter (Cobetter PES membrane filter, China). The resulting filtrates (phage lysates) were stored at 4 °C.

### Concentration and purification of the obtained phages stock lysates

Concentrations of phages were carried out using a method described by Ackermann (2009). Dextran sulfate polyethylene glycol system was used for purification of the phage particles (Othman et al. 2008). The phage suspension was kept at 4 °C for further studies.

### Morphological characterization of phages

The morphology of purified phages was examined by transmission electron microscope. To prepare the phages, 1 mL of pure phage suspension was centrifuged at 20,400 × *g* for 1.5 h at 4 °C and washed twice using phage buffer ((SM buffer): For 1 L: 5.8 g, NaCl; 50 mL, 1 M Tris-HC [pH 7.5]; 2 g, MgSO4.7H2O; 5 mL, 2% gelatin). The supernatant was discarded and the pellet was re-suspended in 20 µL of sterile SM buffer, 5 µL of the re-suspended phages were carried out on a 200-mesh Formvar carbon-coated copper grid and negatively stained with 2% (w/v) phosphotungstic acid (PTA), pH 7.0. The grid was dried for 5 min and then observed at 80 kV using transmission microscope (JEOL-JEM-1010 Electron microscope) at The Regional Center For Mycology and Biotechnology, Al-Azhar University, Egypt.

### Determination of optimal multiplicity of infection (MOI)

The optimum infection multiplicity was described as the ratio of virus component to potential host cells. The bacterial cells formed from their early logarithmic stage of growth (OD_600_ = 0.5, 10^8^ CFU/mL) infected with their particular phage by different ratios (MOI = 0.001, 0.01, 0.1, 1, or 100). After incubation for 12 h at 37 °C, the phage lysate was centrifuged at 4000 × *g* for 15 min. The phage lysate was filtered via a 0.22-μm pore-size syringe filter to evaluate the phage titer. The highest value of phage titers was considered to be the optimal MOI for the phage (Yang et al. 2010).

### Determination of host range and efficiency of plating (EOP)

The host range of each of the isolated phage was tested on other isolates from the same and different genera through the spot test. The results were interpreted after incubation at 37 °C for 24 h by the level of lytic activity as ( +) of phage lysis, ( ±) of weak lysis, and (-) of no lysis. The resulting positive isolates were again tested for their plaque forming ability to calculate plating efficiency (EOP) using a double agar overlay method (Khan Mirzaei and Nilsson 2015).

### One-step growth curve experiment

One-step growth curve experiment was performed according to Pajunen et al. (2000). *E. cloacae* complex 6AS1 was grown at 37 °C to mid-exponential phase (OD_600_ = 0.4–0.5) before being centrifuged at 4000 × *g* for 10 min at 4 °C. The cell pellet was then re-suspended in a fresh Luria–Bertani (LB) broth of 0.1 volumes. A phage suspension of 0.1 mL aliquot was then dispensed into 0.9 mL of bacterial suspension and phages allowed to adsorb for 5 min at 37 °C. The mixture was then centrifuged; pelleted cells were re-suspended in 10 mL LB broth and incubation was continued at 37 °C. Samples were taken at 10-min intervals for 90 min. and immediately diluted and plated for phage titration. Triplicate assays were conducted. The graph was composed of a log_10 _PFU/mL over time. The one-step growth curves of vB_EclM-EP1 and vB_EclM-EP2 propagated on *E. cloacae* complex 6AS1 were determined. The latent period and the burst size of the phages were determined (Buttimer et al. 2018).

### Effects of pH and temperature on phages stability

The phages stability at different pH ranges was tested by suspending the phages in 1 ml of the SM buffer previously calibrated for pH with 1 M NaOH or 1 M HCl at 2, 3, 5, 7, 9, 11, and 12. Phage preparations were incubated at room temperature for 30, 60, and 90 min. Serial dilutions were checked for the lytic effect of the phage in an a double-layer agar against *E. cloacae* complex 6AS1.

Temperature tolerance of phages was tested at 4, 25, 37, 45, 55, 70, and 80 °C, at different intervals of 30, 60, and 90 min in the water bath. After incubation, serial phage dilutions were prepared and phage titers were obtained using the double agar overlay plaque assay (Capra et al. 2006; Hammerl et al. 2014). The assay was done in triplicates and the average titers were determined.

### Extraction and detection of phages nucleic acid

Phages nucleic acids were extracted by using the phenol/chloroform method according to Sambrook and Russell (2001). Phage nucleic acids were treated with DNase I and RNase A, according to the supplier’s instructions (Thermo Scientific). The nucleic acid-treated mixtures were analyzed by electrophoresis at 100 V in a 1.0% agarose gel stained with ethidium bromide using a 1 kb DNA ladder as marker.

### Bacterial reduction assay

The bacteriolytic activity of the phages against the respective host bacterial isolate was checked according to Bibi et al. (2016). Briefly, a 24-h bacterial culture (1 × 10^8^ CFU/mL) and each phage (vB_EclM-EP1 and vB_EclM-EP2) with MOI 0.1 and 0.01 (optimal MOI), respectively, and phage cocktail (vB_EclM-EP1 and vB_EclM-EP2) were inoculated into flasks, while flasks containing the same concentration of bacteria without phages were added as controls. The optical density (600 nm) was taken at intervals of 2 h for a 24 h period after incubation at 37 °C with shaking at 120 rpm in incubator-shaker (Lab-line Instruments, Inc., USA). The ability of the isolated phages vB_EclM-EP1 and vB_EclM-EP2 and phage-cocktail (vB_EclM-EP1 and vB_EclM-EP2) for reduction in *E. cloacae* complex 6AS1 growth was investigated and has been studied by plotting the bacterial growth at OD_600_ against time. Three independent replicates for each treatment were used.

### Determination of phages activities in the yogurt

This experiment was performed according to Soffer et al. (2017) with few modifications. In this method, an overnight bacterial culture of *E. cloacae* complex 6AS1 that correspond to 2 × 10^8^ CFU/mL was used. It was then adjusted to a final concentration 2 × 10^3^ CFU/mL prior to mixing with yogurt. The bacteria-inoculated samples remained in room temperature for 60 min before phages application. Ten milliliters of each phage vB_EclM-EP1, vB_EclM-EP2, and phage cocktail (vB_EclM-EP1 and vB_EclM-EP2) was inoculated into 100 mL yogurt as treatment; the same volume of sterile distilled water was added for the negative control. The same volume of bacterial culture was added for the positive control. Treatments, negative control, and positive control were stored at 4, 25, and 37 °C for 6 days. The counts of viable bacteria cells were determined at 2, 4, and 6 days. Three replicates of 25 g of each package were put in sterile bags and 225 mL sterile peptone water. The bags were hand-mushed for 30 s. The amount of viable bacteria in each package was calculated by plating 0.5 mL of mixture on different MacConkey plates (Oxoid, Thermo Fisher Scientific Inc., UK). The plates were incubated at 37 °C for 24–48 h, and the final bacterial counts (CFU/g) were calculated after counting the colonies.

### Determination of phages activities in the pasteurized milk

This experiment was performed according to Hu et al. (2016) with few modifications. In this method, overnight cultures of bacterial isolate *E. cloacae* complex 6AS1 which were corresponded to a final concentration of 10^5^ CFU/mL were used. Then 5 mL of diluted culture and 5 mL of each phage vB_EclM-EP1, vB_EclM-EP2, and phage cocktail (vB_EclM-EP1 and vB_EclM-EP2) (10^7^ PFU/mL) were inoculated into 40 mL milk as treatment; 45 mL milk combined with 5 mL sterilized LB broth was used as negative control and 45 mL milk combined with 5 mL of bacteria was used as positive control. Treatments, negative control, and positive control were stored at 4, 25, and 37 °C for 5 days. The viable cells of *E. cloacae* complex 6AS1 were counted at days 1, 2, 3, 4, and 5 of storage using MacConkey agar plates after 24–48 h of incubation at 37 °C. The final bacterial counts (CFU/mL) were calculated.

### Statistical analysis

Mean phage counts were expressed in logarithmic units (log_10_) of the Plaque-Forming Units per milliliter (PFU/mL). Mean bacterial counts were expressed in logarithmic units (log_10_) of the number of colony forming units per milliliter (CFU/mL) for milk and per gram (CFU/g) for yogurt samples. The data are presented as the mean ± standard error (SEM). Tests were conducted with paired samples to determine whether there were statistical differences between phage-treated samples and controls. Statistical analysis of SPSS software was carried out (IBM Corp. Published 2011. Windows IBM SPSS Statistics, version 20.0. IBM, Armonk, NY, USA). Statistically significant differences were considered at a *p*-value < 0.05.

## Results

### Isolation and characterization of *E. cloacae* complex phages

Two phages producing clear lytic plaques were isolated from collected sewage water samples using *E. cloacae* complex 6AS1 as permissive and enrichment host. The phages are designated as vB_EclM-EP1 and vB_EclM-EP2.

**Fig. 1:**
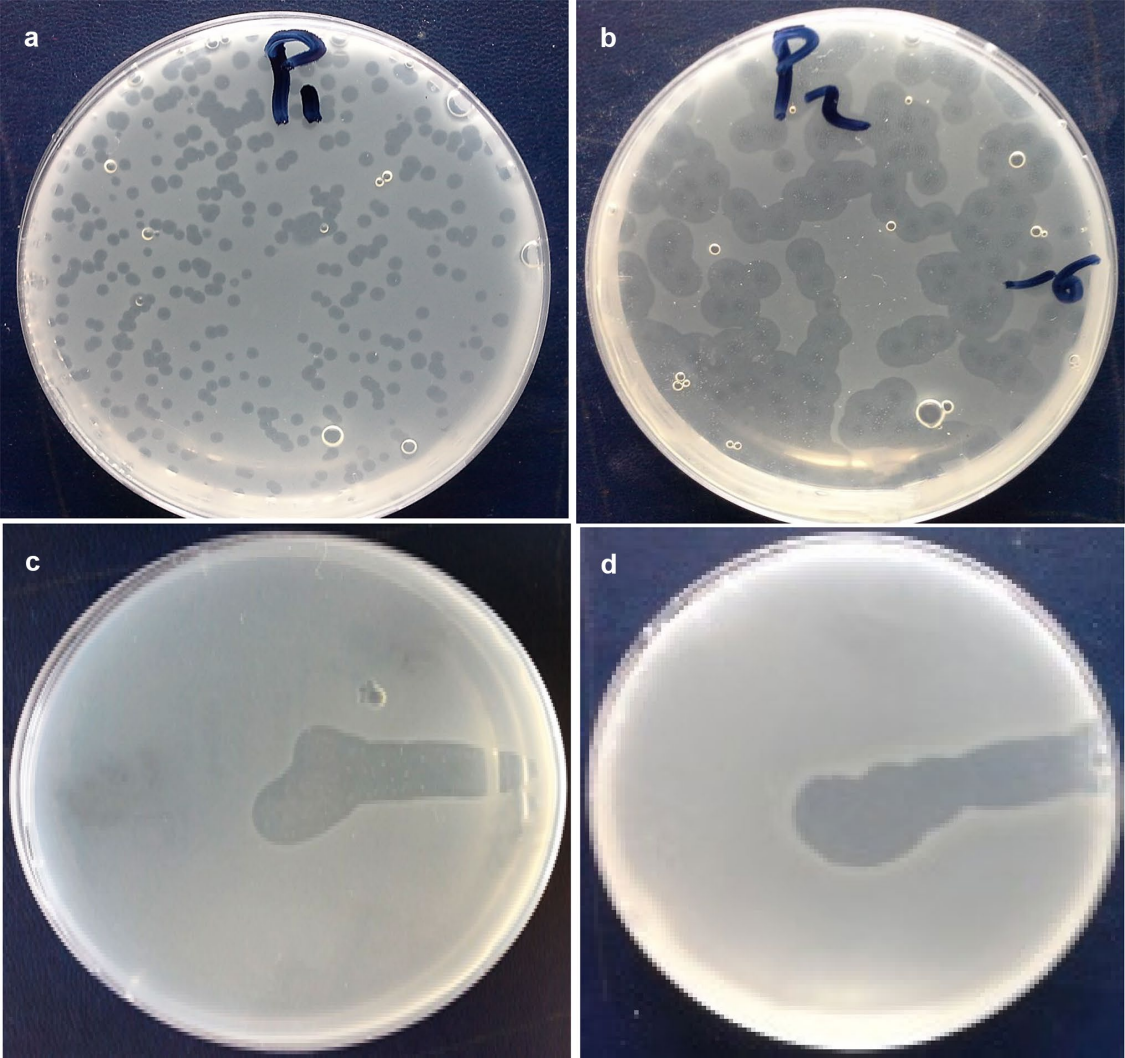
The plaques formed by two phages **a** vB_EclM-EP1 and **b** vB_EclM-EP2 on the lawns *E. cloacae* complex (ECC) and spot test (**c**, **d**) respectively

The plaque morphology of vB_EclM-EP1 appeared small with lytic zone diameter of approximately 1.5 mm and circular, while the plaque of the vB_EclM-EP2 was large with lytic zone diameter of approximately 7 mm, circular, with center and halos (Fig. 1a, b). The lytic zones of the two phages were clear confirming that the isolated phages were virulent (Fig. 1c, d). The virus titer of vB_EclM-EP1 and vB_EclM-EP2 was determined as 3 × 10^8^ and 3.5 × 10^7^ PFU/mL, respectively.

### Morphology of phages

**Fig. 2:**
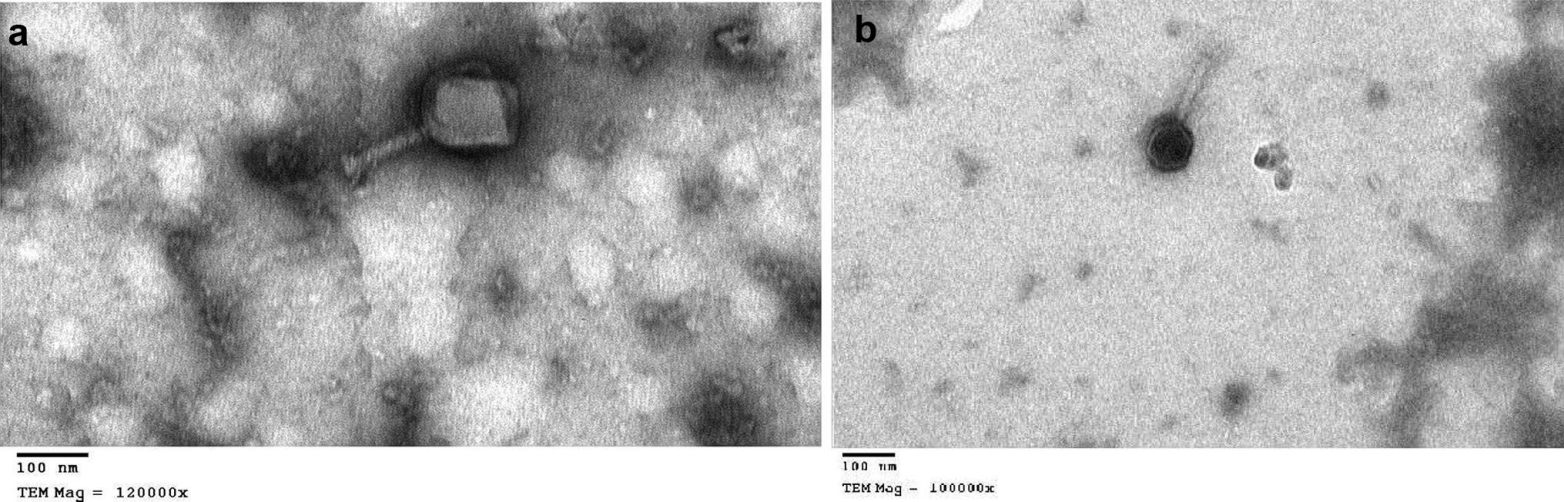
Transmission electron microscopy (TEM) analysis of two phages **a** vB_EclM-EP1 and **b** vB_EclM-EP2. The bar indicates 100 nm

The morphological characteristics and dimensions of the two phages are presented in Table 1. The phages (vB_EclM-EP1 and vB_EclM-EP2) appeared to be composed of contractile tail with an isometric head (Fig. 2a, b). Transmission electron microscopy (TEM) analysis identified that the phages had myoviruses morphology.

**Table 1:** Morphological properties of isolated phages

**Phage**	**Titre (PFU/mL)**	**Total dimension (nm)**	**Capsid length (nm)**	**Capsid diameter (nm)**	**Tail length (nm)**	**Tail diameter (nm)**
vB_EclM-EP1	3 × 10^8^	256.3	130.0	123.6	126.3	20.9
vB_EclM-EP2	3.5 × 10^7^	187.2	73.6	73.6	113.6	18.5

### Host range and efficiency of plating (EOP)

Lytic activity of isolated phages against MDR *E. cloacae* complex isolates (6AS1, 3AS2, 8CS3, and 2AS4) and other bacteria is illustrated in Table 2. Phages vB_EclM-EP1 and vB_EclM-EP2 were able to infect *E. cloacae* complex isolates (6AS1, 3AS2, and 8CS3), while 2AS4 revealed weak lysis. Moreover, vB_EclM-EP1and vB_EclM-EP2 had lytic activity against *Aeromonas hydrophila* and *Klebsiella pneumoniae* which are isolates that belong to different genera. However, no plaque production was observed in *E. cloacae* complex isolate (2AS4), *Staphylococcus aureus*, *Streptococcus* spp., *Proteus* spp., *Salmonella* spp., *Shigella* spp. *Enterobacter* spp., *Pseudomonas oryzihabitans*, *Pseudomonas aeruginosa*, and *Escherichia coli* (Table 2).

**Table 2:** Host range of isolated phages using spot test and EOP

**Isolate**	**Bacterial species**	**Source**^a^	**vB_EclM-EP1**		**vB_EclM-EP2**	
			**Spot test**^**b**^	**Efficiency of plating (EOP)**	**Spot test**	**Efficiency of plating (EOP)**
6AS1	*E. cloacae* complex	This study-(contaminated yogurt)	+	Host	+	Host
3AS2	*E. cloacae* complex	This study-(contaminated milk)	+	0.00006	+	0.004
8CS3	*E. cloacae* complex	This study-(contaminated milk)	+	0.00007	+	0.0009
2AS4	*E. cloacae* complex	This study-(contaminated yogurt)	±	0	±	0
En1	*Enterobacter* spp*.*	Lab isolate	-	0	-	0
Pso1	*Pseudomonas oryzihabitans*	Lab isolate	-	0	-	0
Psa1	*Pseudomonas aeruginosa*	Lab isolate	-	0	-	0
Ec1	*Escherichia coli*	Lab isolate	-	0	-	0
Aeh	*Aeromonas hydrophila*	Lab isolate	+	0.00003	+	0.0002
Klp	*Klebsiella pneumoniae*	Lab isolate	+	0.00003	+	0.0002
Sta1	*Staphylococcus aureus*	Lab isolate	-	0	-	0
St1	*Streptococcus* spp.	Lab isolate	-	0	-	0
Pr1	*Proteus* spp.	Lab isolate	-	0	-	0
Sa1	*Salmonella* spp.	Lab isolate	-	0	-	0
Sh1	*Shigella* spp.	Lab isolate	-	0	-	0

^a^Source of bacterial species in this work was obtained from: This study (contaminated dairy products) or Lab isolate (Microbiology lab, Botany and Microbiology Dept., Faculty of Science, Benha Univ.)

^b^The level of lytic activity was expressed by (+) lysis, (±) weak lysis, and (-) no lysis and efficiency of plating (EOP)

### Optimal multiplicity of infection

Phages vB_EclM-EP1 and vB_EclM-EP2 showed an optimal MOI of 0.1 and 0. 01, respectively.

### One-step growth curve

**Fig. 3:**
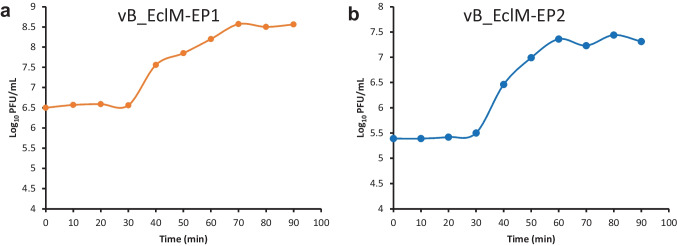
One-step growth curve of two phages **a** vB_EclM-EP1 and **b** vB_EclM-EP2. Data are expressed as means

Phages (vB_EclM-EP1 and vB_EclM-EP2) had burst sizes 100 and 142 PFU/cell respectively with latent period of 30 min (Fig. 3a, b).

## Stability of *E. cloacae* complex phages at different pH and temperature 

**Fig. 4:**
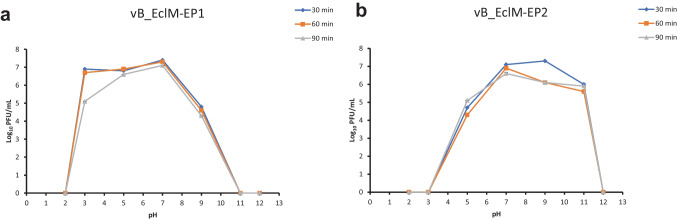
pH stability of two phages **a** vB_EclM-EP1 and **b** vB_EclM-EP2. Results are expressed as means of PFU/mL ± standard error (SEM)

**Fig. 5:**
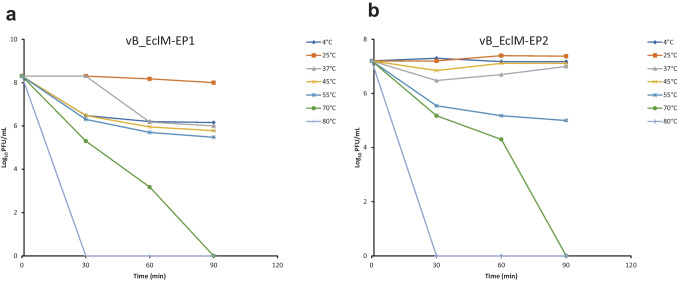
Thermal stability of two phages **a** vB_EclM-EP1 and **b** vB_EclM-EP2. Results are expressed as means of PFU/mL ± standard error (SEM)

Results of pH stability testing revealed that vB_EclM-EP1 was stable at pH ranging 3–9 (Fig. 4a), while vB_EclM-EP2 was sensitive to pH 3 and stable at pH values ranging from 5 to 11 (Fig. 4b). Phage vB_EclM-EP1 was completely inactivated at pH 11. Results obtained from temperature stability assays demonstrated that vB_EclM-EP1 was stable at temperatures 25 °C and 37 °C and its viability decreased at 45 °C and 55 °C (Fig. 5a), while vB_EclM-EP2 remained stable at temperatures ranging from 4 to 45 °C (Fig. 5b). Decrease in infectivity was observed following incubation at 70 °C for 60 min for vB_EclM-EP1 and vB_EclM-EP2, while the two phages were completely inactivated by incubation at 70 °C for 90 min or 80 °C for 30 min.

## Nucleic acid analysis of the isolated *E. cloacae* complex phages

Phages vB_EclM-EP1 and vB_EclM-EP2 nucleic acids were sensitive to DNase I, and did not digest by treatment of RNase A. Therefore, the genomes of isolated vB_EclM-EP1 and vB_EclM-EP2 had double-stranded DNA. The genome sizes were more than 10 kb (Fig. S2).

### Bacterial reduction assay

**Fig. 6:**
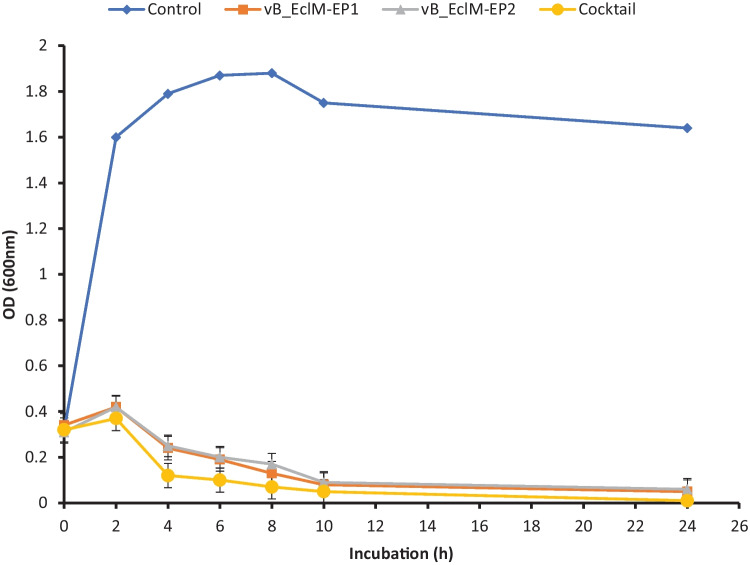
Lytic activity of phages vB_EclM-EP1, vB_EclM-EP2, and their cocktail against *E. cloacae* complex (ECC) in LB medium at an optimal MOI. Absorbance measurements at 600 nm were made every 2 h. The data were expressed as mean ± standard error (SEM)

A gradual OD_600_ decrease was observed in the phage and phage-cocktail-treated bacterial culture compared to the control culture (with no phage). When both phages were applied together as a phage cocktail-treated bacterial culture, a more reduction in *E. cloacae* Complex growth was observed as remarkable reduction in the optical density to reach 0.1 or less within 6 h and was sustained for about 24 h of incubation. Phages and their cocktail stop the growth of *E. cloacae* complex 6AS1 during 24 h. Bacterial optical density continuously increased from 0.3 to over 1.87 for non-infected *E. cloacae* culture during 6 h incubation and showing a normal pattern of growth as shown in Fig. 6.

## Suppression of *E. cloacae* Complex growth in yogurt

**Fig. 7:**
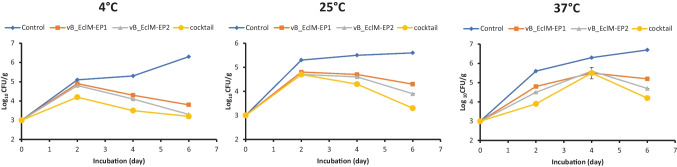
Effect of phages vB_EclM-EP1, vB_EclM-EP2, and their cocktail against *E. cloacae* complex (ECC) in yogurt at different temperatures. The data were expressed as mean ± standard error (SEM)

A reduction in viable cell numbers was detected following the addition of vB_EclM-EP1 and vB_EclM-EP2 and phage cocktail (vB_EclM-EP1 and vB_EclM-EP2) into yogurt (Fig. 7). Without phages infection, *E. cloacae* complex 6AS1 cell counts increased up to around 5.5 log_10_, 5.6 log_10_, and 6.7 log_10_ CFU/g in yogurt treatments at 4, 25, and 37 °C, respectively. With phages infection, cell numbers gradually began to decrease in treatments after 2 days of phage inoculation at 4 °C and 25 °C and 4 days at 37 °C.

At 4 °C, phage vB_EclM-EP1 reduced *E. cloacae* complex 6AS1 counts around 2.5 log_10_ CFU/g after 6 days treatment, while around 3 log_10_ CFU/g, reduction was detected by vB_EclM-EP2 and phage cocktail (vB_EclM-EP1 and vB_EclM-EP2).

At 25 °C, the reduction in viable cell numbers was around 1.3 log_10_ by action of vB_EclM-EP1, and 1.7 and 2.3 log_10_ CFU/g by treatment with vB_EclM-EP2 and phage cocktail (vB_EclM-EP1 and vB_EclM-EP2), respectively, after 6 days of phages infection.

At 37 °C, the reduction in viable cell numbers was around 1.5, 2, and 2.5 log_10_ CFU/g by treatment with vB_EclM-EP1, vB_EclM-EP2, and phage cocktail (vB_EclM-EP1 and vB_EclM-EP2), respectively, after 6 days of phages infection.

## Suppression of *E. cloacae* complex growth in pasteurized milk

**Fig. 8:**
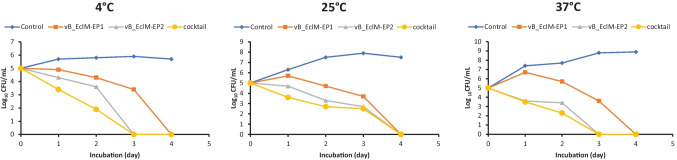
Effect of phages vB_EclM-EP1, vB_EclM-EP2, and their cocktail against *E. cloacae* complex (ECC) in milk at different temperatures. The data were expressed as mean ± standard error (SEM)

The antimicrobial effect of vB_EclM-EP1, vB_EclM-EP2, and phage cocktail (vB_EclM-EP1 and vB_EclM-EP2) at 4, 25, and 37 °C on the milk that was inoculated with *E. cloacae* complex 6AS1 is shown in Fig. 8. Without phages infection, *E. cloacae* complex 6AS1 cell counts increased up to approximately 5.9 log_10_, 7.9 log_10_, and 8.9 log_10_ CFU/mL in milk treatments at 4, 25, and 37 °C, respectively. With phages infection, *E. cloacae* complex 6AS1 cell numbers gradually began to decrease in all treatments after 1 day of phages inoculation.

The growth of *E. cloacae* complex 6AS1 was completely prevented after 3 days of milk treatments with phage vB_EclM-EP2 and phage cocktail (vB_EclM-EP1 and vB_EclM-EP2) at 4 °C and 37 °C, and after 4 days at 25 °C, while phage vB_EclM-EP1 completely inhibited counts of *E. cloacae* complex 6AS1 after 4 days of milk treatments at 4, 25, and 37 °C (Fig. 8).

These results clearly revealed that the vB_EclM-EP1 and vB_EclM-EP2 and phage cocktail (vB_EclM-EP1and vB_EclM-EP2) were active under different storage conditions, killing *E. cloacae* complex 6AS1 not only in milk but also in the yogurt treatment.

## Discussion

This study aimed to isolate *Enterobacter* spp.-specific phages to delay spoilage of dairy products, thus extending shelf-life, and to prevent foodborne illness associated with pathogenic species. This study indicated that these phages had the ability to reduce and prevent the growth of MDR *E. cloacae* complex 6AS1 in yogurt and milk under different storage conditions.

*E. cloacae* complex strains have been isolated from various food sources (Haryani et al. 2008; Maull et al. 2012; Abdel-Hamied et al. 2017). *E. cloacae* is a recognized contaminant in raw and pasteurized milk, cream and in dried milk products, and in dairy products such as ice cream yogurt and cheese samples in Egypt (Sobeih et al. 2020). This contamination has been likely attributed by post-processes such as handling and packaging (Cooney et al. 2011). The above reports coincide with our study where we isolated four MDR *E. cloacae* complex from contaminated packaged milk and yogurt samples within the time of expiry and after expiry date.

*E. cloacae* was reported to be the second most prevalent bacteria from ready-to-eat foods (Nyenje et al. 2012). It is also able to convert lactic acid to propionic and butyric acids leading to an increase in pH in fermented foods (Pérez-Díaz et al. 2019).

Most isolates of these bacteria are complex and resistant to many types of antibiotics (Stock et al. 2001; Bolourchi et al. 2022). In this study, *E. cloacae* complex isolates were found to be MDR. These results have similarity with a study done by Haryani et al. (2008) that found *E. cloacae* isolates were resistant to more than one antibiotic with high multiple antibiotic resistance (MAR) index.

To overcome these bacteria, phage therapy could be one of the best alternative treatments to control bacterial infections in humans and animals, as well as reduce food contamination (Summers 2001; Vikram et al. 2021). The reduction of colonization of bacteria in foods during industrial food manufacturing (biocontrol) can be done by adding phages directly to food surfaces such as animals, fresh items, and foods or even blended to raw milk. For limiting growth of pathogenic and spoilage bacteria on refrigerated foods (especially psychrotrophic bacteria); once the foods are taken to room temperature, phages can further control their proliferation (Bigwood et al. 2008).

Phage plaque morphologies such as size and lysis clarity vary according to several attributes such as the host bacterium used in isolation, geographical diversities of the source of samples, incubation conditions, and percentage of agar concentration used in plating (Jurczak-Kurek et al. 2016; Montso et al. 2019). In this study, two types of distinct plaque morphologies and sizes for *E. cloacae* complex phages were identified. For phage VB_EclM-EP2, we observed plaques with haloes; the haloes could have stemmed from diffusion and the subsequent action of soluble, phage-produced enzymes destroying the cell envelope (Jurczak-Kurek et al. 2016) which concurs with another study done in Kenya on *E. cloacae* phages from environmental waste water (Mutai et al. 2022). Additionally, phage vB_EclM_CIP9 isolated in Canada against clinical *E. cloacae* isolate from a municipal wastewater sample (Wang et al. 2020) and *E. cloacae*-specific phage φ238 was isolated from fermented cucumber sample (Lu et al. 2020).

Phages are very specific to the bacteria they infect; however, a few and rare cross-reactivity to different genera and species have been reported. Lu et al. (2020) detected that *E. cloacae*-phage Φ225 infected two *E. cloacae* strains while phages Φ107, Φ115, and Φ238 infected one *E. cloacae* strain from three *E. cloacae* strains. Interestingly, Φ226 is capable of infecting bacteria in two different genera (*Enterobacter* and *Leclercia*). In this study, the phages vB_EclM-EP1 and VB_EclM-EP2 showed an infection to MDR *E. cloacae* complex isolates (6AS1, 3AS2, and 8CS3) in addition to *Aeromonas hydrophila* isolated from food samples and *Klebsiella pneumoniae* isolated from clinical sample. Phages that are capable of crossing genera may use receptors, intermediary functions, or both, common to a wide range of bacteria (Bielke et al. 2007). On the other hand, Cieślik et al. (2022) reported that phage vB_EclM-45 that was isolated from environmental sample had the highest species specificity causing lysis on various species belonging to *Enterobacter* (*E. cloacae*, *E. hormaechei*, and* E. kobei*).

Phages have unique and distinct morphologies such as capsid, tail, and tail fibers which are different from bacterial cells (Egido et al. 2022). The morphology of the isolated *E. cloacae* complex phages under the transmission electron microscope indicated that they have icosahedral capsids and contractile tails which are descriptive characteristics of members of the order *Caudovirales* (Ackermann 2012; Turner et al. 2021). Similarly, many authors reported that the phages specific for *E. cloacae* complex have an isometric polyhedron (icosahedron) (Nair et al. 2021; Cieślik et al. 2022).

For the identification of nucleic acid type, purified phage genomic DNA was subjected to nuclease treatment using DNase I and RNase A (Wang et al. 2016). The genome of the studied phages was larger than 10 kb; it was not digested by RNase A and we can deduce that the genome is double-stranded DNA. Similarly, Khawaja et al. (2016) showed the genetic material of *E. cloacae* phages TSE1, TSE2, and TSE3 was digested by DNase I and their genomes appeared more than 12 kb in size. According to the size of the genome, the size categorization placed them among medium- to large-sized genomes, which are commonly double-stranded (Abedon 2011). The reported small size phage genomes are around 5.5 kb, while the larger size genomes may be greater than 250 kb (Hatfull 2008).

Multiplication parameters of phage vB_EclM-EP1 and vB_EclM-EP2 were determined using one-step growth curve conditions. The latent period was defined as the time interval between the adsorption and the beginning of the first burst, and the burst size was calculated using the final number of free phage particles to the initial number of phages. vB_EclM-EP1 and vB_EclM-EP2 showed a latent period of about 30 min and burst sizes of 100 and 142 virions per cell, respectively, which are beneficial for biocontrol activities. On the other hand, Jamal et al. (2019) previously reported that *E. cloacae* MJ2 phage had a latent time of 21 min and a very large burst size of 350 virions per cell which is much larger than the burst sizes of the two phages used in this study while *Enterobacter* virus myPSH1140 had a very short latency period of 11 min and a burst size of 135 virions per cell (Manohar et al. 2019). Life cycle parameters of phages will play important role in determining both in vitro and in vivo phage activities (during therapy), because phage multiplication is directly proportional to reduction in bacteria (Manohar et al. 2019).

Due to the dairy environment characterized by wide range of pH (from 4.5 to 6.8) and temperatures (from 4 to 121 °C), it is necessary to determine the environmental infectivity of newly isolated phages for use as biocontrol agents in dairy products. An example of the industrial use of *E. cloacae*-specific phages is the attempt to use them as factors for reduction of the bloating defect caused by these bacteria in cucumber fermentation (Jończyk et al. 2011). In pH studies, the two *E. cloacae* phages showed relatively good stability at pH ranging from 5 to 9; moreover, vB_EclM-EP2 was resistance to a more alkaline environment at pH (11). Similar tolerance to a wide pH range was also shown by three new phages (Entb_43, Entb_44, and Entb_45) with a pH ranging from 4 to 11 (Cieślik et al. 2022). In the same context, five *E. hormaechei* virulent phages (Ehp-YZU08, Ehp-YZU10, Ehp-YZU9-1, Ehp-YZU9-2, and Ehp-YZU9-3) which were isolated from sewage in China were able to survive in a pH range of 5–10 (Chen et al. 2022). The ability of phages to tolerate at varying pH levels indicates that they may be suitable for administration through the food preservation.

On temperature studies, the *E. cloacae* phages survived at all the tested temperatures from 4 to 45 °C; however, the phage titer decreased when treated at 55 to 70 °C and reach complete reduction after 90 min incubation at 70 °C. A previous study by Khawaja et al. (2016) reported the optimal temperature of TSE phages of *E. cloacae*; however, no lytic activity was observed after treatment at 65 °C. Another study was achieved by Jamal et al. (2019) which reported that the phage MJ2 of *E. cloacae* was stable at temperatures ranging from 37 to 70°C and lost its activity at 80 °C. Our results regarding the inactivation are in confirmation with those observed by Basdew and Laing (2014) who reported that increasing in temperature decreases virus survival and activity.

The usage of phages in phage therapy can be estimated by the effects of the bacterial growth reduction study (Haq et al. 2012). The bacterial growth control experiment suggested that the capacity of the phage in incubation time reduced the growth of the target bacteria during 24 h post phages infection compared to control. These findings are almost similar to a study done by Khawaja et al. (2016), who reported that the TSE phages of *E. cloacae* reduced the growth of the target bacteria at 8 h post infection until 18 h, after which resistant bacterial cells started to emerge.

The efficacy of phage as food preservative agent depended on various factors, such as food matrix, surface area, contact time, structure, bacterial load, dose of phage, and presence of other compounds (García-Anaya et al. 2020; Ly-Chatain 2014). In the dairy industry, phage treatment has included the control of pathogens in different dairy products (Sillankorva et al. 2012). The decrease in viable count of *E. cloacae* complex cell counts in the yogurt following the treatment with vB_EclM-EP1 and vB_EclM-EP2 phages infection shows the potential use of phages in food industry. After 6 days of vB_EclM-EP2 treatment, the high rate of bacterial reduction was achieved (3 log_10_ CFU/g) at a temperature 4 °C thus because vB_EclM-EP2 was more stable and still had a high titer compared to room temperature storage (37 °C); the high rate of reduction occurred due to the active growth of *E. cloacae* complex at 4 °C as psychrotrophs (Cousin 1982; Hu et al. 2016). On the contrary, at a temperature 4 °C, the viable bacterial cells were inactivated, and phage requires an active growth phase of bacteria for replication (Rogovski et al. 2021). At room temperature, Soffer et al. (2017) obtained reductions of about 0.07, 0.26, and 1.01 log_10_ CFU/g for *Shigella sonnei* by applying three different concentrations (5, 6, and 7 log_10_ PFU, respectively) of a commercial phage (ShigaShieldTM) to contaminated yogurt.

At different storage temperature (4, 25, and 37 °C), the antimicrobial effect of isolated phages was detected in packaged milk that was inoculated with *E. cloacae* complex 6AS1. vB_EclM-EP2 and phage cocktail (vB_EclM-EP1 and vB_EclM-EP2) completely eradicated *E. cloacae* complex 6AS1 after 3 days of incubation, while vB_EclM-EP1 totally inhibited *E. cloacae* counts after 4 days of milk treatment. The application of the phage cocktail (SPHG1 and SPHG3) at MOIs of 1000 or 100 resulted in a significant decrease in the viable count of *S*. *typhimurium* by 4.2 log_10_/sample in milk and water (Esmael et al. 2021). Garcia et al. (2009) used a cocktail of two phages (Φ5 and Φ72) against *Staphylococcus aureus* in different types of milk, including ultra-high temperature (UHT), pasteurized, whole, and semi-skimmed milk. These authors found bacterial reductions of 1.0 to 3.6 log_10_ CFU/mL.

The basic mechanisms involved in the phage infection process in complex systems such as milk and dairy products still need to be clarified. In spite of this, the effectiveness of phages decreased in yogurt than in milk and this may be attributed to the organic substances such as proteins, fat content, inhibitory substances, and the conditions of food matrix and viscosity (Hosseinidoust et al. 2014). Moreover, yogurt had a greater surface area than milk, which resulted in less interaction between phages and their host bacteria and a few phages may have been inhibited by yogurt additives or yogurt provided additional defense layer for the bacteria (Soffer et al. 2017; García-Anaya et al. 2020).

## Conclusions

This is the first report on the effectiveness of specific phages to control the MDR *E. cloacae* complex growth in milk and yogurt. These results demonstrate that lytic *E. cloacae* complex-specific phages isolated from sewage have high pH and thermal tolerance and have the capacity to infect different *E. cloacae* complex isolates. For phages to be used for biocontrol purposes, there is need to study their physical and chemical traits such as different storage and pH conditions to optimize their lytic activity. Lytic phages have a great potential in food and dairy industry in inhibiting and elimination of pathogenic bacteria.

### Supplementary Information

Below is the link to the electronic supplementary material.

Supplementary file1 (DOCX 546 KB)

## References

[CR1] Abdel-Hamied MR, Salha GD, Deabes M (2017). Isolation, characterization and antibiotics susceptibility of β-glucuronidase producing *Escherichia coli* and other enteric bacteria from ground beef. Afr J Biotechnol.

[CR2] Abedon ST (2011) Size does matter-distinguishing bacteriophages by genome length (and ‘breadth’). Microbiol Aust 32:95–96. 10.1071/MA11095

[CR3] Ackermann HW (2009) Phage classification and characterization. In Bacteriophages. Methods and Protocols, Vol. I, Isolation, Characterization, and Interactions (Clokie MRJ, Kropinski AM, eds). Method Mol Biol 501:127–140. Humana Press, Clifton, NJ. 10.1007/978-1-60327-164-6_13 10.1007/978-1-60327-164-6_13 19066817

[CR4] Ackermann HW (2012). Bacteriophage electron microscopy. Adv Virus Res.

[CR5] Adams MH (1959). Bacteriophages.

[CR6] Annavajhala MK, Gomez-Simmonds A, Uhlemann A-C (2019). Multidrug-resistant *Enterobacter cloacae* complex emerging as a global, diversifying threat. Front Microbiol.

[CR7] Bacon RT, Belk KE, Sofos JN, Clayton RP, Reagan JO, Smith GC (2000). Microbial populations on animal hides and beef carcasses at different stages of slaughter in plants employing multiple-sequential interventions for decontamination. J Food Prot.

[CR8] Basdew IH, Laing MD (2014). Stress sensitivity assays of bacteriophages associated with *Staphylococcus aureus*, causal organism of bovine mastitis. African J Microbiol.

[CR9] Bibi Z, Abbas Z, Rehman S (2016). A phage P.E1 isolated from hospital sewage reduces the growth of *Escherichia coli*. Biocontrol Sci Technol.

[CR10] Bielke L, Higgins S, Donoghue A, Donoghue D, Hargis BM (2007). *Salmonella* host range of bacteriophages that infect multiple genera. Poult Sci.

[CR11] Bigwood T, Hudson JA, Billington C, Carey-Smith GV, Heinemann JA (2008). Phage inactivation of foodborne pathogens on cooked and raw meat. Food Microbiol.

[CR12] Bolourchi N, Giske CG, Nematzadeh S, Mirzaie A, Abhari SS, Solgi H, Badmasti F (2022). Comparative resistome and virulome analysis of clinical NDM-1–producing carbapenem-resistant *Enterobacter cloacae* complex. J Glob Antimicrob Resist.

[CR13] Boor KJ, Murphy SC (2005) Microbiology of market milks. In: Robinson RK (ed.): Dairy Microbiology Handbook: The Microbiology of Milk and Milk Products. New Jersey, John Wiley & Sons: 91–122. 10.1002/0471723959.ch3

[CR14] Brüssow H (2005). Phage therapy: the *Escherichia coli* experience. Microbiol.

[CR15] Buttimer C, Lucid A, Neve H, Franz C, O’mahony J, Turner D,  (2018). *Pectobacterium atrosepticum* phage vB_PatP_CB5: a member of the proposed genus *‘Phimunavirus’*. Viruses.

[CR16] Capra ML, Quiberoni A, Reinheimer J (2006). Phages of *Lactobacillus* casei/paracasei: response to environmental factors and interaction with collection and commercial strains. J Appl Microbiol.

[CR17] Chen CW, Yuan L, Zhang YS, Mgomi FC, Wang Y, Yang ZQ, Jiao XA (2022) Comparison of biological and genomic characteristics of five virulent bacteriophages against *Enterobacter hormaechei*. Microb Pathog 162:105375. 10.1016/j.micpath.2021.105375 10.1016/j.micpath.2021.105375 34974119

[CR18] Cieślik M, Harhala M, Orwat F, Dąbrowska K, Górski A, Jończyk-Matysiak E (2022) Two newly isolated Enterobacter-specific bacteriophages: biological properties and stability studies. Viruses 14:1518. 10.3390/v14071518 10.3390/v14071518 PMC9319786 35891499

[CR19] CLSI (2018) Performance standards for antimicrobial susceptibility testing. 28th ed. CLSI supplement M100. Wayne, PA

[CR20] Cooney S, Lversen C, Healy B, O'Brien S Fanning S (2011) *Enterobacter* spp. Elsevier Ltd. 72–80

[CR21] Cousin MA (1982). Presence and activity of psychrotrophic microorganisms in milk and dairy products: a review 1. J Food Prot.

[CR22] Cristobal-Cueto P, García-Quintanilla A, Esteban J, García-Quintanilla M (2021). Phages in food industry biocontrol and bioremediation. Antibiotics.

[CR23] Davin-Regli A, Masi M, Bialek S, Nicolas-Chanoine MH, Pagès JM, Li X-Z, Elkins CA, Zgurskaya HI (2016). Antimicrobial resistance and drug efflux pumps in *Enterobacter* and *Klebsiella*. Efflux-mediated drug resistance in bacteria: mechanisms, regulation and clinical implications.

[CR24] De Smet J, Hendrix H, Blasdel BG (2017). Pseudomonas predators: understanding and exploiting phage–host interactions. Nat Rev Microbiol.

[CR25] Egido JE, Costa AR, Aparicio-Maldonado C, Haas PJ, Brouns SJJ (2022) Mechanisms and clinical importance of bacteriophage resistance. FEMS Microbiol Rev 46:fuab048. 10.1093/femsre/fuab048 10.1093/femsre/fuab048 PMC8829019 34558600

[CR26] El-Dougdoug NK, Cucic S, Abdelhamid AG, Brovko L, Kropinski AM, Griffiths MW, Anany H (2019). Control of *Salmonella* Newport on cherry tomato using a cocktail of lytic bacteriophages. Int J Food Microbiol.

[CR27] Esmael A, Azab E, Gobouri AA, Nasr-Eldin MA, Moustafa MMA, Mohamed SA, Badr OAM, Abdelatty AM (2021). Isolation and characterization of two lytic bacteriophages infecting a multi-drug resistant *Salmonella* typhimurium and their efficacy to combat salmonellosis in ready-to-use foods. Microorganisms.

[CR28] Farmer JJ, Asbury MA, Hickman FW, Brenner DJ (1980). *Enterobacter sakazakii*: a new species of “*Enterobacteriaceae*” isolated from clinical specimens. Int J Syst Bacteriol.

[CR29] Fernandes R (2009) Microbiology handbook: dairy products. 3rd Ed. Cambridge. R Soc Chem 1–15:182

[CR30] Frank JF (1997) Milk and dairy products. Food Microbiology – Fundamental and Frontiers (Doyle P, Beuchat R & Montville J, eds), 169–186. ASM Press, Washington, DC

[CR31] Garcia P, Madera C, Martinez B, Rodriguez A, Suarez JE (2009). Prevalence of bacteriophages infecting *Staphylococcus aureus* in dairy samples and their potential as biocontrol agents. J Dairy Sci.

[CR32] García-Anaya MC, Sepulveda DR, S´aenz-Mendoza AI, Rios-Velasco C, Zamudio- Flores PB, Acosta-Mu˜niz, CH,  (2020). Phages as biocontrol agents in dairy products. Trends Food Sci Technol.

[CR33] Hammerl J A, Jäckel C, Alter T, Janzcyk P, Stingl K, Knüver MT et al (2014) Reduction of *Campylobacter jejuni* in broiler chicken by successive application of group ii and group iii phages*. *PLoS ONE 9:e114785. 10.1371/journal.pone.0114785 10.1371/journal.pone.0114785 PMC4260947 25490713

[CR34] Haq IU, Chaudhry WN, Andleeb S, Qadri I (2012). Isolation and partial characterization of a virulent bacteriophage IHQ1 specific for *Aeromonas punctata* from stream water. Microb Ecol.

[CR35] Haryani Y, Tunung R, Chai LC, Lee HY, Tang SY, Son R (2008). Characterization of *Enterobacter cloacae* isolated from street foods. Asean Food J.

[CR36] Hatfull GF (2008). Bacteriophage genomics. Curr Opin Microbiol.

[CR37] Hosseinidoust Z, Olsson AL, Tufenkji N (2014). Going viral: designing bioactive surfaces with bacteriophages. Colloids Surf B Biointerfaces.

[CR38] Hu ZY, Meng XC, Liu F (2016). Isolation and characterisation of lytic bacteriophages against *Pseudomonas* spp., a novel biological intervention for preventing spoilage of raw milk. Int Dairy J.

[CR39] Hudson JA, Billington C, Carey-Smith G, Greening G (2005). Bacteriophages as biocontrol agents in food. J Food Prot.

[CR40] Jamal M, Andleeb S, Jalil F, Imran M, Nawaz MA (2019). Isolation, characterization and efficacy of phage MJ2 against biofilm forming multi-drug resistant *Enterobacter cloacae*. Folia Microbiol.

[CR41] Jończyk E, Kłak M, Międzybrodzki R, Górski A (2011). The influence of external factors on bacteriophages. Folia Microbiol.

[CR42] Jurczak-Kurek A, Gąsior T, Nejman-Faleńczyk B, Bloch S, Dydecka A, Topka G, Necel A, Jakubowska-Deredas M, Narajczyk M, Richert M, Mieszkowska A, Wróbel B, Węgrzyn G, Węgrzyn A (2016). Biodiversity of bacteriophages: morphological and biological properties of a large group of phages isolated from urban sewage. Sci Rep.

[CR43] Khan Mirzaei M, Nilsson AS (2015) Isolation of phages for phage therapy: a comparison of spot tests and efficiency of plating analyses for determination of host range and efficacy. PLoS ONE 10:e0118557. 10.1371/journal.pone.0118557 10.1371/journal.pone.0118557 PMC4356574 25761060

[CR44] Khawaja AK, Abbas Z, Rehman US (2016). Isolation and characterization of lytic phages TSE1-3 against *Enterobacter cloacae*. Open Life Sci.

[CR45] Liu S, Tang Y, Wang D, Lin N, Zhou J (2016). Identification and characterization of a new *Enterobacter* onion bulb decay caused by *Lelliottia amnigena* in China. Appli Micro Open Access.

[CR46] Lu Z, Pérez-Díaz IM, Hayes JS, Breidt F (2020). Bacteriophages infecting gram-negative bacteria in a commercial cucumber fermentation. Front Microbiol.

[CR47] Ly-Chatain MH (2014). The factors affecting effectiveness of treatment in phages therapy. Front Microbiol.

[CR48] Manohar P, Tamhankar AJ, Lundborg CS, Nachimuthu R (2019). Therapeutic characterization and efficacy of bacteriophage cocktails infecting *Escherichia coli*, *Klebsiella pneumoniae*, and *Enterobacter* species. Front Microbiol.

[CR49] Maull KD, Hickey ME, Lee JL (2012) The study and identification of bacterial spoilage species isolated from catfish during refrigerated storage. J Food Process Technol S11–003. 10.4172/2157-7110.6AS11-003

[CR50] Mezzatesta ML, Gona F, Stefani S (2012). *Enterobacter cloacae* complex: clinical impact and emerging antibiotic resistance. Future Microbiol.

[CR51] Mohammed AS, Abdullahi M (2015). Comparative study of microbial quality of hawked nono and packaged yogurt sold in Bida. Spec J Psychol Manag.

[CR52] Montso PK, Mlambo V, Ateba CN (2019). Characterization of lytic bacteriophages infecting multidrug-resistant Shiga toxigenic atypical *Escherichia coli* O177 strains isolated from cattle feces. Front Public Health.

[CR53] Morozova V, Jdeed G, Kozlova Y, Babkin I, Tikunov A, Tikunova NA (2021). New *Enterobacter cloacae* bacteriophage EC151 encodes the deazaguanine DNA modification pathway and represents a new genus within the Siphoviridae family. Viruses.

[CR54] Moye Z, Woolston J, Sulakvelidze A (2018). Bacteriophage applications for food production and processing. Viruses.

[CR55] Mutai IJ, Juma AA, Inyimili MI, Nyachieo A, Nyamache AK (2022) Efficacy of diversely isolated lytic phages against multi-drug resistant *Enterobacter cloacae* isolates in Kenya. Afr J Lab Med 11:a1673. 10.4102/ajlm.v11i1.1673 10.4102/ajlm.v11i1.1673 PMC9453119 36091354

[CR56] Nair A, Vyawahare R, Khairnar K (2021). Characterization of a novel, biofilm dispersing, lytic bacteriophage against drug-resistant *Enterobacter cloacae*. J Appl Microbiol.

[CR57] Nale JY, Clokie MR (2021). Preclinical data and safety assessment of phage therapy in humans. Curr Opin Biotechnol.

[CR58] Nascimento ECD, Sabino MC, Corguinha LDR, Targino BN, Lange CC, Pinto CLO, Pinto PF, Vidigal PMP, Sant'Ana, AS, Hungaro HM (2022) Lytic bacteriophages UFJF_PfDIW6 and UFJF_PfSW6 prevent *Pseudomonas fluorescens* growth *in vitro* and the proteolytic-caused spoilage of raw milk during chilled storage. Food Microbiol 101:103892. 10.1016/j.fm.2021.103892 10.1016/j.fm.2021.103892 34579852

[CR59] Nyenje ME, Odjadjare CE, Tanih NF, Green E, Ndip RN (2012). Foodborne pathogens recovered from ready-to-eat foods from roadside cafeterias and retail outlets in Alice, Eastern Cape Province, South Africa: public health implications. Int J Environ Res Public Health.

[CR60] Othman BA, Askora AA, Awny NM, Abo-Senna ASM (2008). Characterization of virulent bacteriophages for *Streptomyces griseoflavus* isolated from soil. Pak J Biotechnol.

[CR61] Pajunen M, Kiljunen S, Skurnik M (2000). Bacteriophage phiYeO3-12, specific for *Yersinia enterocolitica* serotype O:3, is related to coliphages T3 and T7. J Bacteriol.

[CR62] Pérez-Díaz IM, Hayes J, Medina E, Webber AM, Butz N, Dickey AN (2019). Assessment of the non-lactic acid bacteria microbiota in fresh cucumbers and commercially fermented cucumber pickles brined with 6% NaCl. Food Microbiol.

[CR63] Rogovski P, Cadamuro RD, da Silva R, de Souza EB, Bonatto C, Viancelli A, Michelon W, Elmahdy EM, Treichel H, Rodríguez-Lázaro D Fongaro G (2021) Uses of bacteriophages as bacterial control tools and environmental safety indicators. Front Microbiol 12:793135. 10.3389/fmicb.2021.793135. 10.3389/fmicb.2021.793135 PMC8670004 34917066

[CR64] Sambrook J, Russell DW (2001). Molecular cloning: a laboratory manual.

[CR65] Sillankorva SM, Oliveira H, Azeredo J (2012) Bacteriophages and their role in food safety. Int J Microbiol 863945. 10.1155/2012/863945 10.1155/2012/863945 PMC3536431 23316235

[CR66] Sobeih A, Al-Hawary I, Khalifa E, Ebied N (2020) Prevalence of *Enterobacteriaceae* in raw milk and some dairy products. Kafrelsheikh Vet Med J 18:9–13. 10.21608/KVMJ.2020.39992.1009

[CR67] Soffer N, Woolston J, Li M, Das C, Sulakvelidze A (2017) Bacteriophage preparation lytic for *Shigella* significantly reduces *Shigella sonnei* contamination in various foods. PLoS ONE 12:e0175256. 10.1371/journal.pone.0175256 10.1371/journal.pone.0175256 PMC5376334 28362863

[CR68] Sofos JN, Smith GC (1998). Nonacid meat decontamination technologies: model studies and commercial applications. Int J Food Microbiol.

[CR69] Stephan R, Van Trappen S, Cleenwerck I, Iversen C, Joosten H, De Vos P, Lehner A (2008). *Enterobacter pulveris* sp. nov., isolated from fruit powder, infant formula and an infant formula production environment. Int J Syst Evol Microbiol.

[CR70] Stock I, Grüger T, Wiedemanna B (2001). Natural antibiotic susceptibility of strains of the *Enterobacter cloacae* complex. Int J of Antimicrob Agents.

[CR71] Summers WC (2001). Bacteriophage therapy. Annu Rev Microbiol.

[CR72] Tan CW, Rukayadi Y, Hasan H, Abdul-Mutalib N-A, Jambari NN, Hara H, Thung TY, Lee E, Radu S (2021) Isolation and characterization of six Vibrio parahaemolyticus lytic bacteriophages from seafood samples. Front Microbiol 12:616548. 10.3389/fmicb.2021.616548. 10.3389/fmicb.2021.616548 PMC7987779 33776954

[CR73] Turner D, Kropinski AM, Adriaenssens EM (2021). A roadmap for genome-based phage taxonomy. Viruses.

[CR74] Vikram A, Woolston J, Sulakvelidze A (2021) Phage biocontrol applications in food production and processing. Curr Issues Mol Biol 40:267–302. 10.21775/cimb.040.267 10.21775/cimb.040.267 32644048

[CR75] Wang K, Tamayo MG, Penner TV, Cook BWM, Court DA, Theriault SS (2020). Characterization of the *Enterobacter* phage vB_EclM_CIP9. Microbiol Resour Announc.

[CR76] Wang Z, Zhao X (2022). The application and research progress of bacteriophages in food safety. J App Microbiol.

[CR77] Wang Z, Zheng P, Ji W, Fu Q, Wang H, Yan Y, Sun J (2016). SLPW: a virulent bacteriophage targeting methicillin-resistant *Staphylococcus aureus* in vitro and in vivo. Front Microbiol.

[CR78] Wójcicki M, Błazejak S, Gientka I, Brzezicka K (2019) The concept of using bacteriophages to improve the microbiological quality of ˙minimally processed foods. Acta Sci Pol Technol Aliment 18:373–383. 10.17306/J.AFS.0695 10.17306/J.AFS.0695 31930789

[CR79] Yang H, Liang L, Lin S, Jia S (2010). Isolation and characterization of a virulent bacteriophage AB1 of *Acinetobacter baumannii*. BMC Microbiol.

