# Correction to: Isolation, characterization, and application of lytic bacteriophages for controlling Enterobacter cloacae complex (ECC) in pasteurized milk and yogurt

**DOI:** 10.1007/s12223-023-01067-7

**Published:** 2023-06-06

**Authors:** Mohamed A. Nasr-Eldin, Eman Gamal, Mahmoud Hazza, Sabah A. Abo-Elmaaty

**Affiliations:** Department of Botany and Microbiology, Faculty of Science, Benha University, Benha, 13511 Egypt

**Correction to: Folia Microbiologica** **https://doi.org/10.1007/s12223-023-01059-7**

The original version of the Supplementary Information file unfortunately contained an error. The Supplementary Information file was published without accepting track changes. The corrected Word file with accepted track changes for Supplementary data has been provided.

The original Supplementary Information file has been corrected.

## Supplementary Information

Below is the link to the electronic supplementary material.

Supplementary file1 (DOCX 536 KB)

