# Supplementary material for: Isolation, characterization, and application of lytic bacteriophages for controlling Enterobacter cloacae complex (ECC) in pasteurized milk and yogurt
Source: Folia Microbiol (Praha). 2023 May 15;68(6):911–24. doi: 10.1007/s12223-023-01059-7 (PMC10689537; doi:10.1007/s12223-023-01059-7)
Supplement: Supplementary file 1 — Supplementary file1 (DOCX 546 KB) [file 12223_2023_1059_MOESM1_ESM.docx]

**“Supplementary data”**

**Isolation, characterization, and application of lytic bacteriophages for controlling *Enterobacter cloacae* complex (ECC) in pasteurized milk and yogurt**

Mohamed A. Nasr-Eldin*, Eman Gamal, Mahmoud Hazza and Sabah A. Abo-Elmaaty

Department of Botany and Microbiology, Faculty of Science, Benha University, Benha 13511, Egypt

***Corresponding author:** Mohamed A. Nasr-Eldin

**E-mail:** [mohamed.nasreldin@fsc.bu.edu.eg](mailto:mohamed.nasreldin@fsc.bu.edu.eg)

**Table S1** Biochemical characterization representing four *E. cloacae* complex isolates.

| **Test** | ***E. cloacae* complex (ECC)** | | | |
| --- | --- | --- | --- | --- |
|  | **6AS1** | **3AS2** | **8CS3** | **2AS4** |
| Catalase | + | + | + | + |
| Oxidase | - | - | - | - |
| Glucose fermentation | + | + | + | + |
| Lactose fermentation | - | + | - | + |
| Sucrose fermentation | + | + | + | + |
| Maltose fermentation | + | + | + | + |
| D-sorbitol fermentation | + | + | - | + |
| Indole Production | + | + | + | + |
| Methyl Red | - | - | - | - |
| Voges – Proskauer | + | + | + | + |
| Urease | - | + | + | - |

(+) positive result; (-) negative result

**Table S2** Antibiotic sensitivity pattern of the *E. cloacae* complex isolates against a selection of twelve antibiotics according to standard antibiogram (CLSI, 2018).

| **Antibiotic Class** | **Antibiotic Name** | **Conc. µg** | ***E. cloacae* complex (ECC) isolates** | | | |
| --- | --- | --- | --- | --- | --- | --- |
|  |  |  | *E. cloacae* complex 6AS1 | *E. cloacae* complex 3AS2 | *E. cloacae* complex 8CS3 | *E. cloacae* complex 2AS4 |
| Penicillins | Penicillin (P) | 10 | R | R | R | R |
|  | Amoxicillin (AX) | 25 | R | R | R | R |
| Cephalosporins | Cephalexin (CN) | 10 | S | S | S | S |
|  | Cefotaxime (CT) | 30 | R | R | R | R |
|  | Ceftazidime (CAZ) | 30 | R | R | R | R |
| Monobactam | Aztreonam (ATM) | 30 | R | R | R | R |
| Quinolones | Ciprofloxacin (CIP) | 5 | I | R | S | S |
|  | Norfloxacin (NOR) | 10 | S | S | S | S |
| Aminoglycosides | Amikacin (AK) | 30 | S | S | S | S |
| Glycopeptides | Vancomycin (VA) | 30 | R | R | R | R |
| Chloramphenicol | Chloramphenicol (C) | 30 | S | S | S | R |
| Nitroheterocyclics | Nitrofurantoin (F) | 300 | S | I | R | R |

*R, resistant; I, intermediate; and S, susceptible


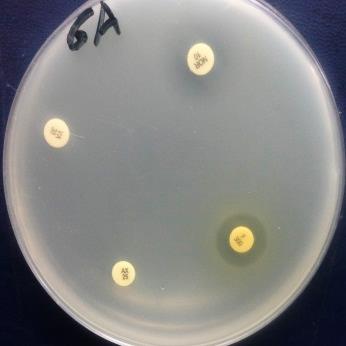

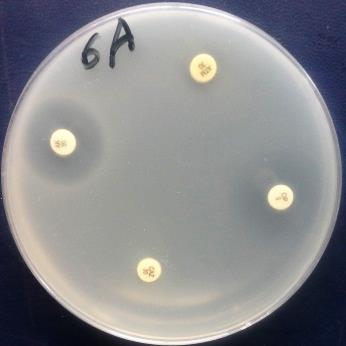

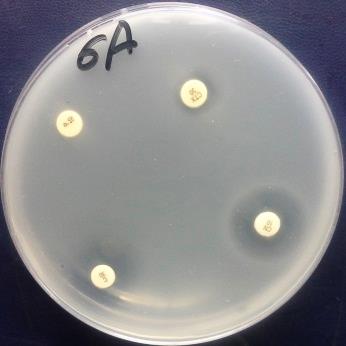


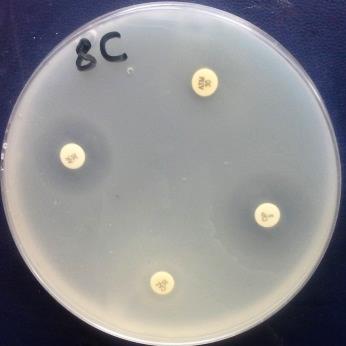

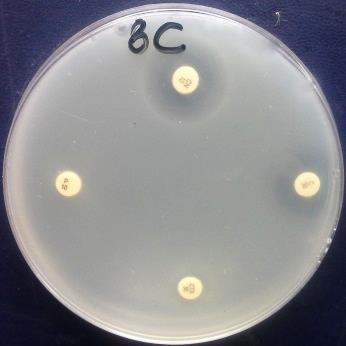

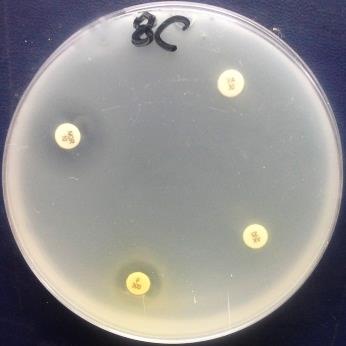


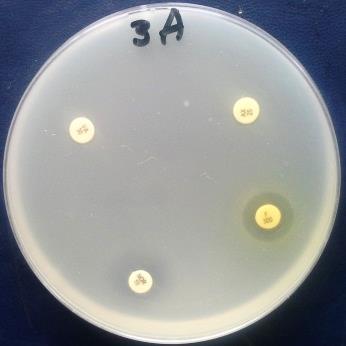

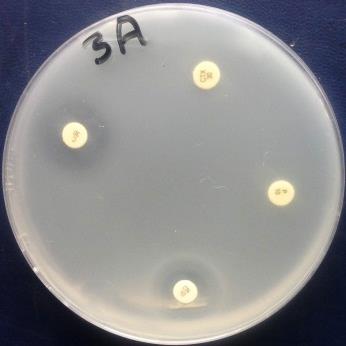

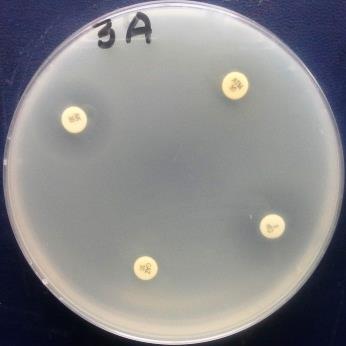


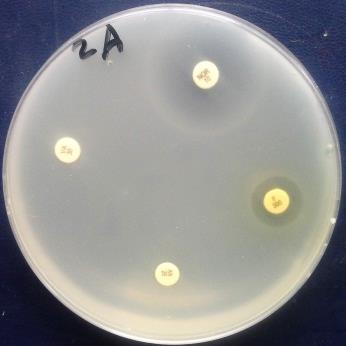

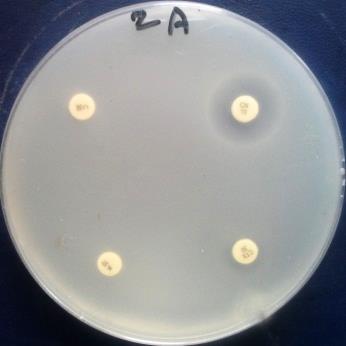

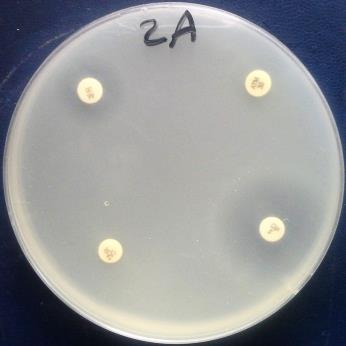


**Fig. S1** Antibiotic susceptibility for *E. cloacae* complex (ECC) isolates.


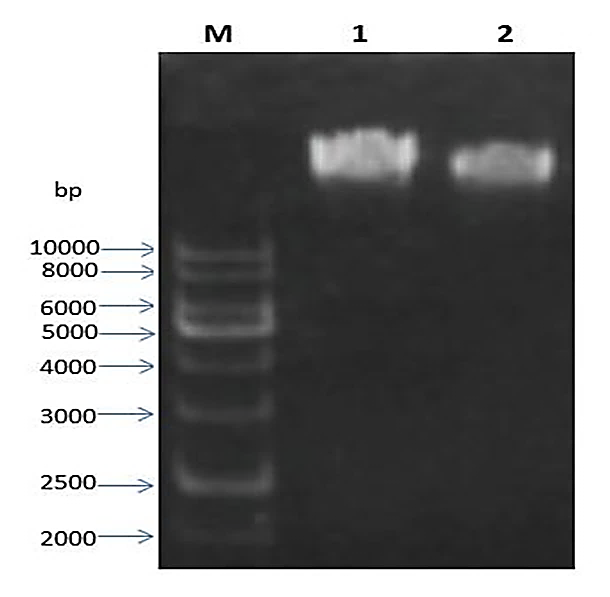


**Fig. S2** Genomic DNA isolated from phages vB_EclM-EP1 and vB_EclM-EP2, treated with RNase A and run on an agarose gel. Lane M: 1-kb DNA marker; lane1: vB_EclM-EP1; lane 2: vB_EclM-EP2.
